# The protective effect of Papaverine and Alprostadil in rat testes after ischemia and reperfusion injury

**DOI:** 10.1590/S1677-5538.IBJU.2017.0600

**Published:** 2018

**Authors:** Mehmet Ali Karagoz, Omer Gokhan Doluoglu, Hatice Ünverdi, Berkan Resorlu, Mehmet Melih Sunay, Arif Demirbas, Tolga Karakan, Arif Aydin

**Affiliations:** 1 Department of Urology, Ankara Training and Research Hospital, Ankara, Turkey; 2 Deparment of Pathology Ankara Training Research Hospital, Ankara, Turkey; 3 Department of Urology, Faculty of Medicine, Canakkale Onsekiz Mart University, Canakkale, Turkey; 4 Saglik Bakanligi Ankara Egitim ve Arastirma Hastanesi Ankara, Ankara, Turkey; 5 Department of Urology, Faculty of Medicine, Necmettin Erbakan University Meram, Konya, Turkey

**Keywords:** Papaverine, Alprostadil, Spermatic Cord Torsion

## Abstract

**Objective:**

To investigate the effect of papaverine and alprostadil on testicular torsion-detorsion injury in rats.

**Materials and Methods:**

A total of 40 male Wistar-Albino rats were used in this study. Four hours of right testicular torsion was applied to each group, excluding sham oper- ated group. The torsion-detorsion (T/D), T/D + papaverine and T/D + alprostadil groups received saline, papaverine and alprostadil at the same time as surgical detorsion, respectively. At 14 days after the surgical detorsion, ischaemic changes and the degree of damage were evaluated with Cosentino scoring and the Johnson tubular biopsy score (JTBS).

**Results:**

JTBS was determined as 8.8±2.7 in the Sham group, 5.08±1.9 in the T/D+papaverine group, 5.29±2.3 in the T/D +alprostadil group and 2.86±1.9 in the TD group. The JTBS was determined to be statistically significantly high in both the T/D + papaverine group and the T/D + alprostadil group compared to the T/D group (p=0.01, p=0.009). In the T/D + papaverine group, 3 (43%) testes were classified as Cosentino 2, 3 (43%) as Cosentino 3 and 1 (14%) as Cosentino 4. In the T/D +alprostadil group, 5 (50 %) testes were classified as Cosentino 2, 3 (30 %) as Cosentino 3 and 2 (20%) as Cosentino 4.

**Conclusion:**

The present study indicated that spermatic cord administration of alprostadil and papaverine showed a protective effect against ischemia/reperfusion injury after right-side testes torsion and histological changes were decreased after testicular ischemia reperfusion injury.

## INTRODUCTION

Testicular torsion is an urological emergency that may result in ischemia and necrosis of the testes. If not recognized in time, torsion may lead to ischemic injury and loss of the testes (1–3). Testicular damage varies depending on the grade and duration of the torsion (4).

To ensure blood flow, surgical detorsion is required in all cases. Reperfusion of the ipsi-lateral testes after ischemia results in overproduction of free oxygen radicals (FORs), cytokines, and adhesion molecules, in addition to the migration of leukocytes (5, 6). The main patho physiology of testicular torsion/detorsion (T/D) seems to be ischemia/reperfusion (I/R) injury of the testes. FORs and cytokines are major initiating components of the testicular injury. The administration of vasodilator drugs has potential antioxidant and therapeutic effects on I/R injury of the testes (7). To the best of our knowledge, no study has investigated the role of alprostadil in testicular I/R injury and only one study could be found in literature related to the protective effect of papaverine on testicular I/R injury. The aim of this study was to investigate the protective role of papaverine and alprostadil in testicular I/R injury.

## MATERIALS AND METHODS

Following approval from the Local Ethics Committee (No: 4/5/15-367) a total of 40 male Wistar-Albino rats, each weighing 250-300 grams, were included in the study. All rats were fed with standard laboratory rat chow and water ad libitum on a 12 hour light/dark cycle at 21-24°C. All animals were handled in compliance with the recommendations of the animal care committee of the university and the Principles of Laboratory Animal Care (NIH publication No: 85-23, revised 1985).

Rats were randomly divided into 4 groups as follows: sham operated, torsion/detorsion group (T/D), T/D+papaverine group, and T/D+alprostadil group. The rats were anesthetized with the administration of 50mg/kg ketamine hydrochloride and 10mg/kg xylazine hydrochloride under aseptic conditions.

In the sham group (n=10), the right testes was removed through the incision and then replaced in the scrotum. In this group, no T/D procedure was performed. In the T/D group (n=10), saline (2mL of 0.9% NaCl) was injected by using 30 gauge needle into the spermatic cord at the same time as detorsion was applied. In the T/D+papaverine group (n=10), 2mL of papaverine (20mg/kg) was injected by using 30 gauge needle into the last 1cm part of the spermatic cord at the same time as detorsion. In the T/D+alprostadil (20μg/kg) group (n=10), 2mL of alprostadil was injected by using 30 gauge needle into the last 1cm of spermatic cord at the same time as detorsion. Torsion was induced by twisting the right-side testis 720 in a clockwise direction and this position was maintained for 4 hours by fixing the testes to the scrotum with 4/0 polyglactin (Vicryl; Ethicon Inc, Johnson&Johnson Co., Somerville, NJ, USA). At the end of the testicular ischemia period, the testes were released and restored to the normal position to allow reperfusion. After this operation, the rats were fed for 14 days and were then euthanised and right orchiectomy was performed. In the 14 day postoperative care period, 3 rats died in the T/D+papaverine group, so examination could only be made of 7 rats in that group.

The testicular tissue was fixed in 10% formaldehyde for histological analyses. Tissue sections of 4 micrometer thickness were stained with hematoxylin-eosin and evaluated by an experienced genitourinary pathologist. Ischaemic changes in the testes and the degree of damage were evaluated with Cosentino scoring (8) and the Johnson tubular biopsy score (JTBS). In Co-sentino scoring, the testis is classified in 4 grades. Grade 1 to 4 stands for normal testis parenchyma to coagulation necrosis in the parenchyma. JTBS was developed to examine spermatogenesis histopatologically after testicular damaging circumstances. A Johnson score of 9 or 10 represents normal histology, a score of 8 hyposperma-togenesis, 3-7 maturation arrest, 2 germinal cell aplasia and a score of 1, tubular fibrosis (9).

The data analysis was performed using SPSS for Windows, version 23.0 (SPSS Inc., Chicago, IL, USA). Conformity to normality of distribution was tested with P-P plot and Kolmogorov-Smirnov tests. Kruskal Wallis variance analysis was used for intergroup comparisons of continuous variables (Post hoc: Bonferroni), and the Chi square test was used for the comparison of categorical variables. Descriptive statistics for variables with non-normal distribution and nominal variables were presented as median (min-max) and the number of cases (n) and percentage (%), respectively. A value of p<0.05 was considered statistically significant.

## RESULTS

**Figure 1:**
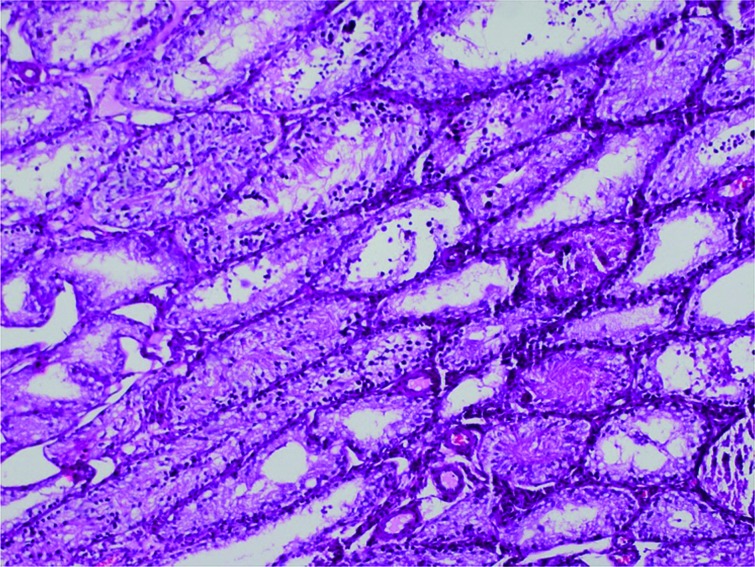
Histopathology image of the testis parenchyma obtained from the papaverine group (Group 3) (Cosentino Grade 2).

In the histopathological evaluation of removed right testes, 9 (90%) testes in the sham group were determined as Cosentino Grade 1, and 1 as Grade 4. In the T/D group, 5 (50%) testes were Cosentino Grade 3 and 5 (50%) were Grade 4. In the T/D+papaverine group, 3 (43%) testes were Cosentino Grade 2 (Figure-1), 3 (43%) were Grade 3 and 1 (14%) was Grade 4.[Fig f2]

**Figure 2 f2:**
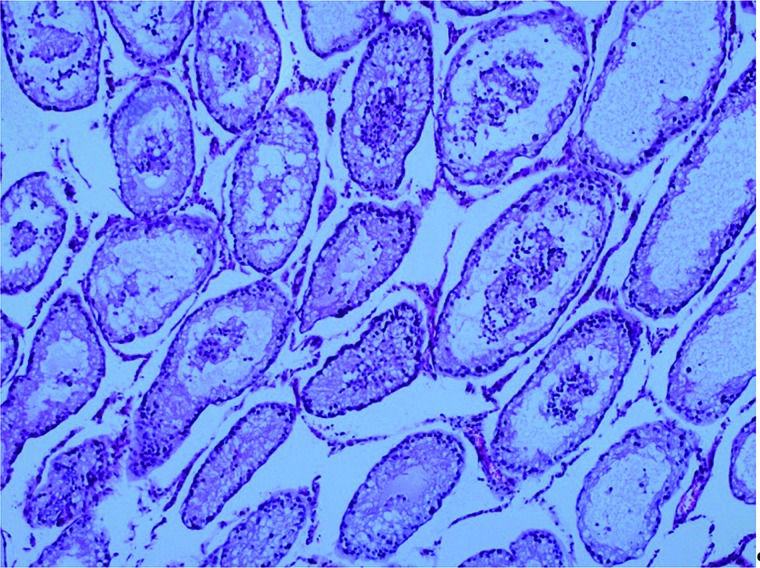
Histopathology image of the testis parenchyma obtained from the alprostadil group (Group 5) (Cosentino Grade 3).

**Figure 3:**
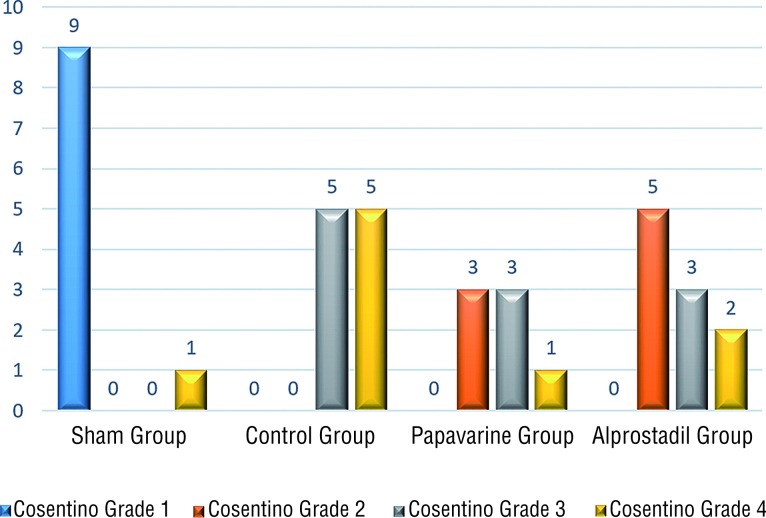
Distribution of the groups according to Cosentino grades.

In the T/D+alprostadil group, 5 (50%) testes were Cosentino Grade 2, 3 (30%) were Grade 3 (Fi-gure-2) and 4 (20%) were Grade 4 (Figure-3).

The Cosentino scores of the T/D+papaverine group were determined to be better than those of the T/D group but the difference was not statistically significant (p=0.55). The Cosentino histopa-thological findings of the T/D+alprostadil group were observed to be significantly better than those of the T/D group (p=0.03). The Cosentino grades of the papaverine and alprostadil groups were seen to be similar (p=0.85).

**Figure 4:**
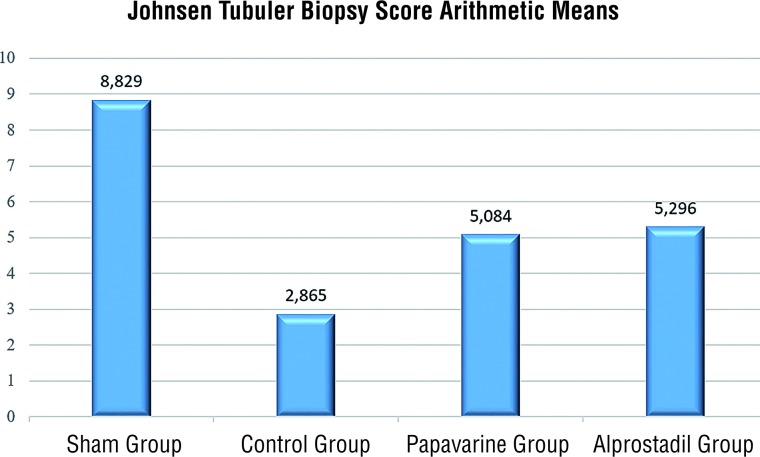
Distribution of the arithmetic means of the Johnsen Tubuler Biopsy Scores of the groups.

The JTBS was determined as 5.08±1.9 in the T/D+papaverine group, 5.29±2.3 in the T/D+alprostadil group and 2.86±1.9 in the T/D group. The JTBS was determined to be statistically significantly high in both the T/D+papaverine group and the T/D+alprostadil group compared to the T/D group (p=0.01, p=0.009) (Figure-4).

## DISCUSSION

Testicular torsion is a medical emergency seen in males, particularly in adolescence, but also in childhood and young adulthood (10). To prevent loss of testicular function, surgical detorsion must be applied as an emergency in the treatment. It is necessary to obtain regular testicular blood flow to be able to continue normal spermatogenesis. Hypoxia is the main reason for testicular damage following testicular torsion (11). Although detorsion (reperfusion) provides correction of the blood flow, it can lead to additional dangers for the testis and this is known as reperfusion injury (12–15).

Reperfusion injury results in the formation of toxic FORs together with increased blood flow after ischaemia (12, 16). At the same time, a local and systemic inflammatory response develops. The complementary activation of leukocyte migration and platelet-leukocyte aggregation causes an increase in microvascular permeability (17–19). As a result of these events, testicular damage progresses further. To protect the testis from all forms of oxidative stress and I/R damage, many chemical agents have been tested in experimental studies. Despite successful results, it has still not been possible for these agents to come into clinical use as their use has not been possible on humans because of severe side-effects (7, 20).

Papaverine is an opium alkaloid used in the treatment of vasospasm and erectile dysfunction (15). Papaverine has also been used by direct application to the blood vessels as a smooth muscle relaxant in microsurgery. By inhibiting phosphodiesterase enzyme, the adenosine monophosphate (cAMP) level is usually increased. Another important effect which is formed is a change in mitochondrial respiration (14, 21). Some researchers have stated that sildenafil and other phos-phodiesterase inhibitors may have anti-inflammatory properties by inhibiting ROS, leukocyte infiltration and inflammatory cytokines such as IL-1, IL-6 and TNF (22, 23).

The effect of alprostadil, which is a PGE1 analog, is shown through an increase in adenosine monophosphate (cAMP) with adenylate cyclase activation and by lowering the intracellular Ca++ level. With this effect, alprostadil leads to relaxation in the smooth muscle wall. Alprostadil provides an immunosuppressive and cytoprotective effect with a reduction in leukocyte aggregation and a decrease in TNF expression. Increasing levels of TNF, P-selectin, E-selectin, ICAM-1 and VCAM-1 in ischaemia and reperfusion are significantly reduced with a PGE1 infusion (24). Therefore, activation and degranulation of macrophages and granulositis decrease. In transplanted pulmonary tissue, PGE1 has been shown to reduce the hypothermic thermal effect of ischaemia reperfusion damage and provide protection by increasing the acquisition of glucose and oxygen (25). This effect of PGE1 applied during reperfusion has been shown to be achieved by reducing pro and anti-inflammatory cytokine levels.

In previous studies that have evaluated ischaemia-reperfusion injury in different organs with papaverine and alprostadil, the drugs have been administered intravenously, intra-arterially or intraperitoneally. In the current study, considering that the dilatation effect could be greater in vascular structures, it was thought appropriate to administer papaverine and alprostadil directly into the spermatic cord.

For the histopathological evaluation of testicular deterioration in this study, Cosentino scoring and JTBS were used. In Cosentino scoring, the testis is classified in 4 grades. While Grade 1 shows normal testis parenchyma, Grade 4 testis shows coagulation necrosis in the parenchyma. Johnson et al. developed the JTBS to be able to evaluate spermatogenesis quantitatively following any event which has caused damage to the testis, and this is the currently most widely used scoring system in routine applications. This score gives a score for each seminiferous tubule in the biopsy material and the mean value is calculated for the examined tubules. A Johnson score of 9 or 10 represents normal histology, a score of 8 hypospermatogenesis, 3-7 maturation arrest, 2 germinal cell aplasia and a score of 1, tubular fibrosis (9).

In the revision of literature, it was found that protective effects of alprostadil were not evaluated before, by injection directly into spermatic cord, in a testicular T/D model. In the sham group, 9 testes were evaluated as Cosentino Grade 1 and 1 as Grade 4. The coagulation necrosis seen in 1 was probably related to artery damage during surgery. In 50% of the rats ap-plied with alprostadil, there was a mild degree of deterioration in the alignment of germ cells in the seminiferous tubules and a loss of cohesion in the cells (Grade 2). Only 2 of the testes were Cosentino Grade 4. A significant degree of improvement was observed in the mean JTBS in the alprostadil group compared with the control group (p=0.009).

In the revision of literature, it was found that only one study which has attempted to determine whether or not papaverine has a protective effect on I/R injury in the testes; 35 rats were separated into 5 groups (control, sham-operated, verapamil-treated, lidocaine-treated and papave-rine treated). In that study, papaverine did not show any protective effect in respect of sperm count, motility or morphology (26). In contrast, in the current study, the JTBS of the papaverine group was determined to be significantly higher than that of the T/D group. Cosentino Grade 2 was determined in 3 (43%) rats of this group. Although a histopathological improvement was seen compared to the T/D group, the difference was not statistically significant. However, as the evaluation of this group was made on 7 rats, there could be Type II error in the findings.

## CONCLUSIONS

In the results of the current study, a significant improvement was observed in the JTBS with the application of both papaverine and alprostadil. Protective effects were determined histopathologically in the Cosentino grades with both drugs, although papaverine was not statisti-cally significant. According to these results, these drugs applied to the spermatic cord during de-torsion in rats have a useful, protective effect on testicular injury. To clarify the protective effects of these drugs, there is a need for further studies using different doses and different administration pathways.
